# Determining the Efficacy of Various Governmental Interventions in Stemming the Spread of SARS-CoV-2 in Seven Locations

**DOI:** 10.3389/fpubh.2022.828090

**Published:** 2022-05-06

**Authors:** Jordan Mendelson, Andrew Moawad, Lauren Tesoriero, Marc Wilkenfeld

**Affiliations:** ^1^New York University Long Island School of Medicine, Mineola, NY, United States; ^2^Department of Medicine, New York University Langone Hospital–Long Island, Mineola, NY, United States

**Keywords:** COVID-19, intervention, SARS-CoV-2, masks, social distancing, stay-at-home

## Introduction

In December of 2019, reports emerged of a new pneumonia-like illness emanating from Wuhan, China (1). The cause was determined to be the novel coronavirus SARS-CoV-2, which later instigated the greatest pandemic of the 21st century to date.

Early in the pandemic, local and statewide governments across the United States implemented various interventions intended to stem the spread of SARS-CoV-2 within their jurisdictions. As much was still unknown about SARS-CoV-2 at the time, these interventions were sometimes met with skepticism. As local and statewide governments instituted social distancing guidelines, mask mandates, and stay-at-home orders, the scientific justification behind these was often either hypothesized or unknown. As social distancing and mask wearing have become more engrained within our culture, it is still somewhat unclear to what degree they actually stem the spread of SARS-CoV-2.

More than 12 months into the COVID-19 pandemic, there have been more than 27.7 million confirmed cases and more than 485,000 deaths attributed to COVID-19 within the United States alone (2). While most public health experts agree that governmental interventions help mitigate the spread of SARS-CoV-2, there are others that disagree with this notion, often pointing to the lack of scientific and biostatistical evidence behind such claims. In this study, we attempted to determine the efficacy of various governmental interventions in stemming the spread of SARS-CoV-2 in seven locations.

## Methods

**Figure 1:**
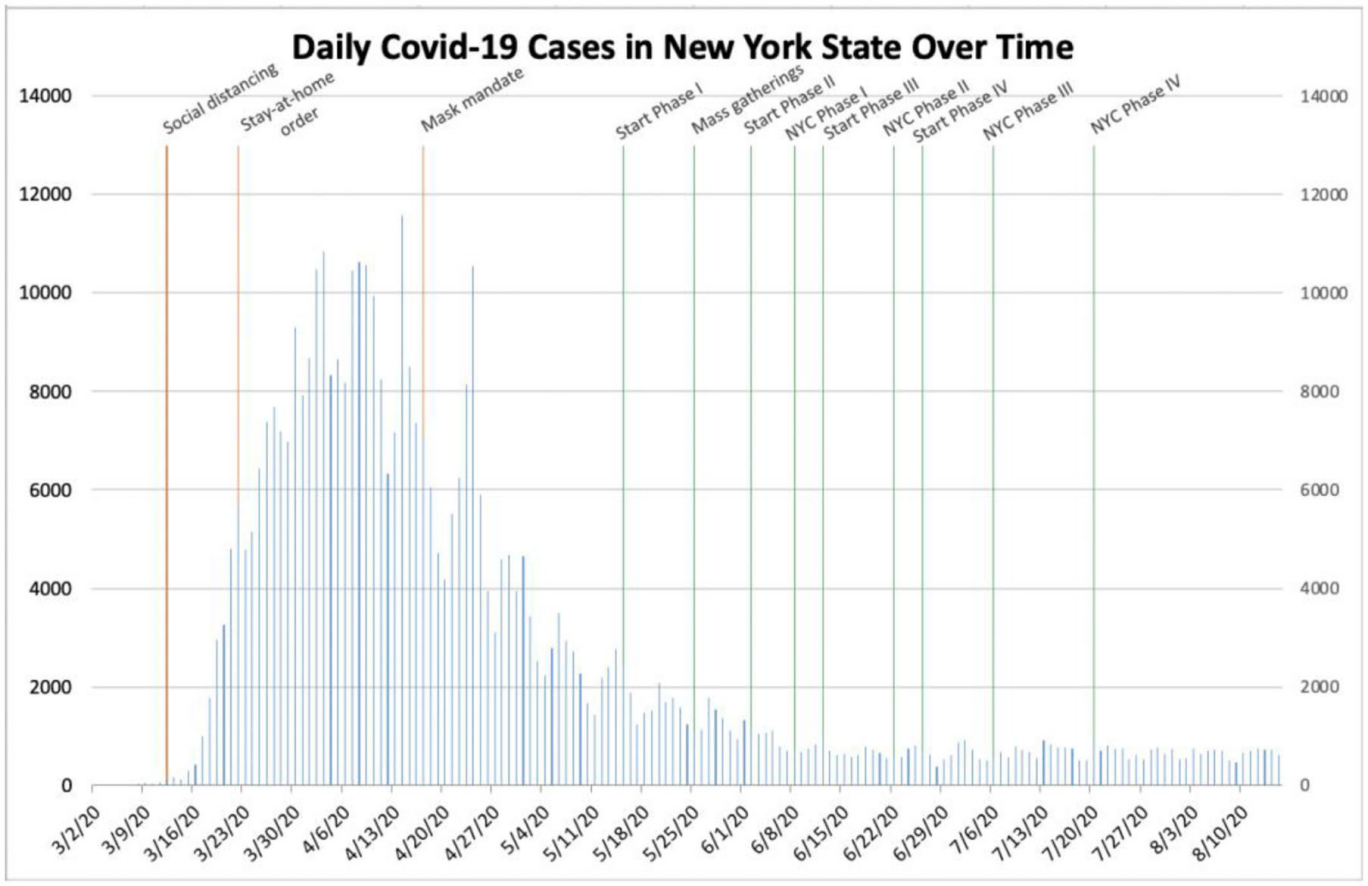
Daily COVID-19 cases in New York State.

We first collected data pertaining to the number of daily cases of COVID-19 within New York State from the beginning of March to August 15, 2020 (3). New York was chosen due to both our location within the state and New York City's distinction as an early epicenter of COVID-19 within the United States. We plotted the dataset as an epidemiological curve and subsequently superimposed various governmental interventions onto it (Figure 1). Due to the incubation period of SARS-CoV-2, estimated by the Centers for Disease Control and Prevention to be 2 to 14 days (4), we hypothesized that the initial effect of each intervention would likely be observed after approximately 14 days. We analyzed the curve with careful inspection.

**Figure 2:**
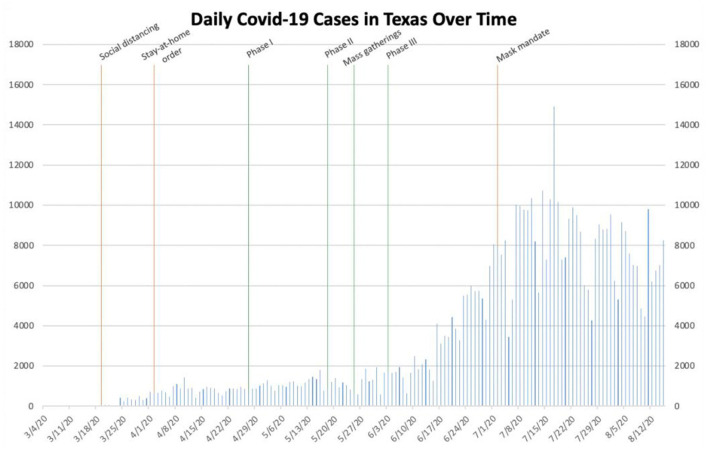
Daily COVID-19 cases in Texas.

**Figure 3:**
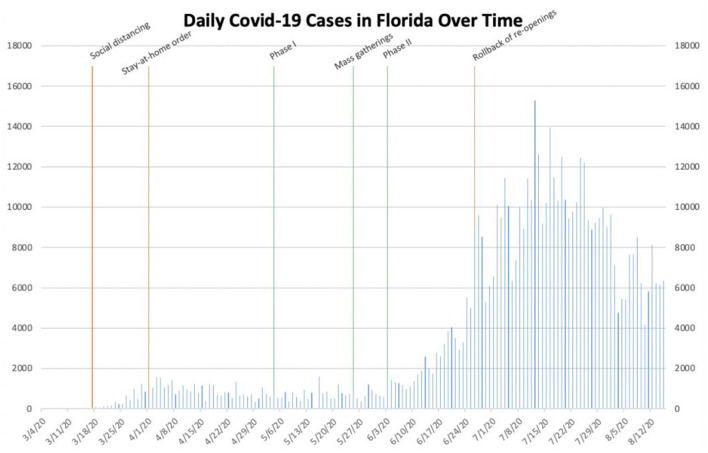
Daily COVID-19 cases in Florida.

**Figure 4:**
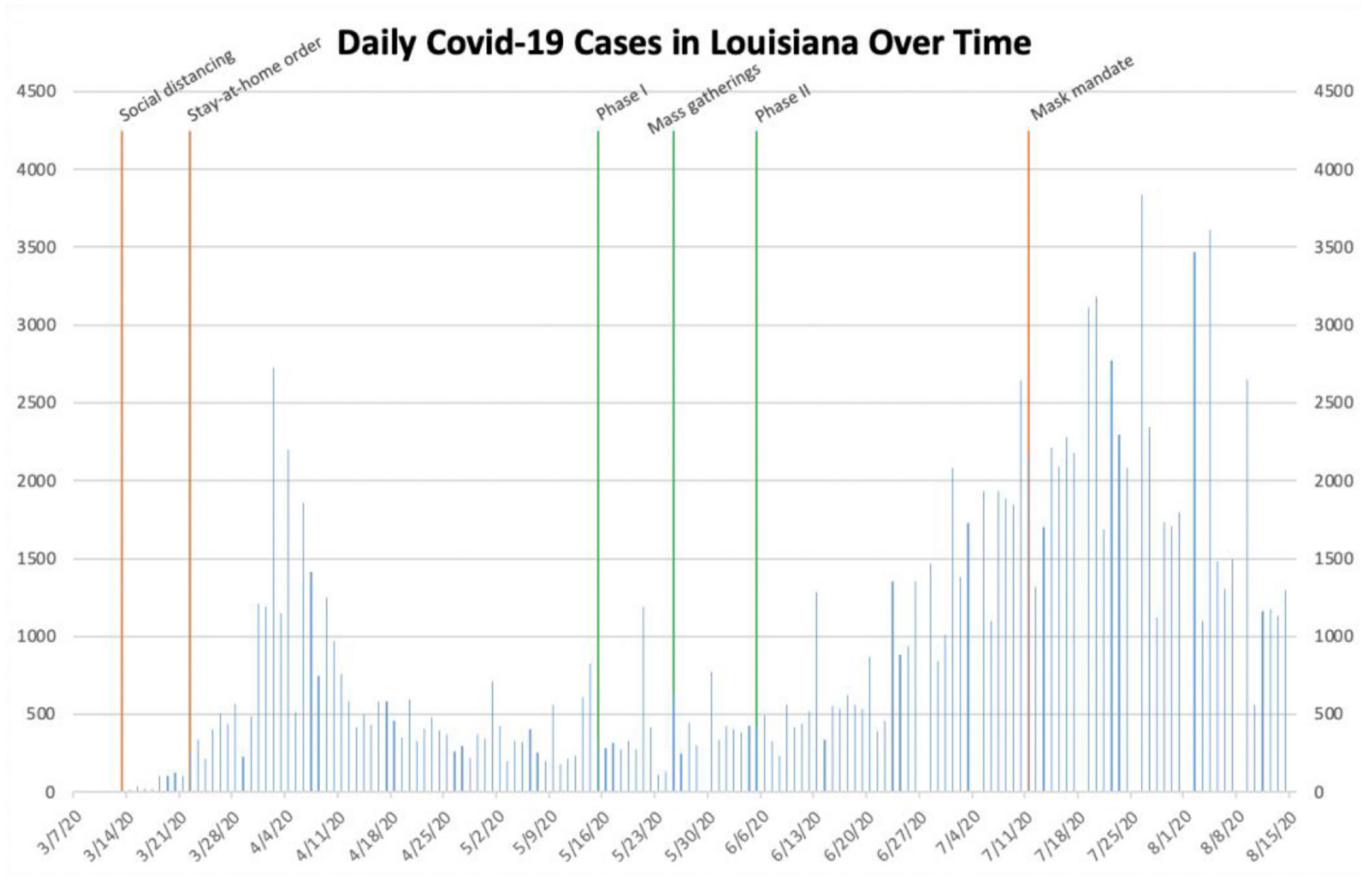
Daily COVID-19 cases in Louisiana.

**Figure 5:**
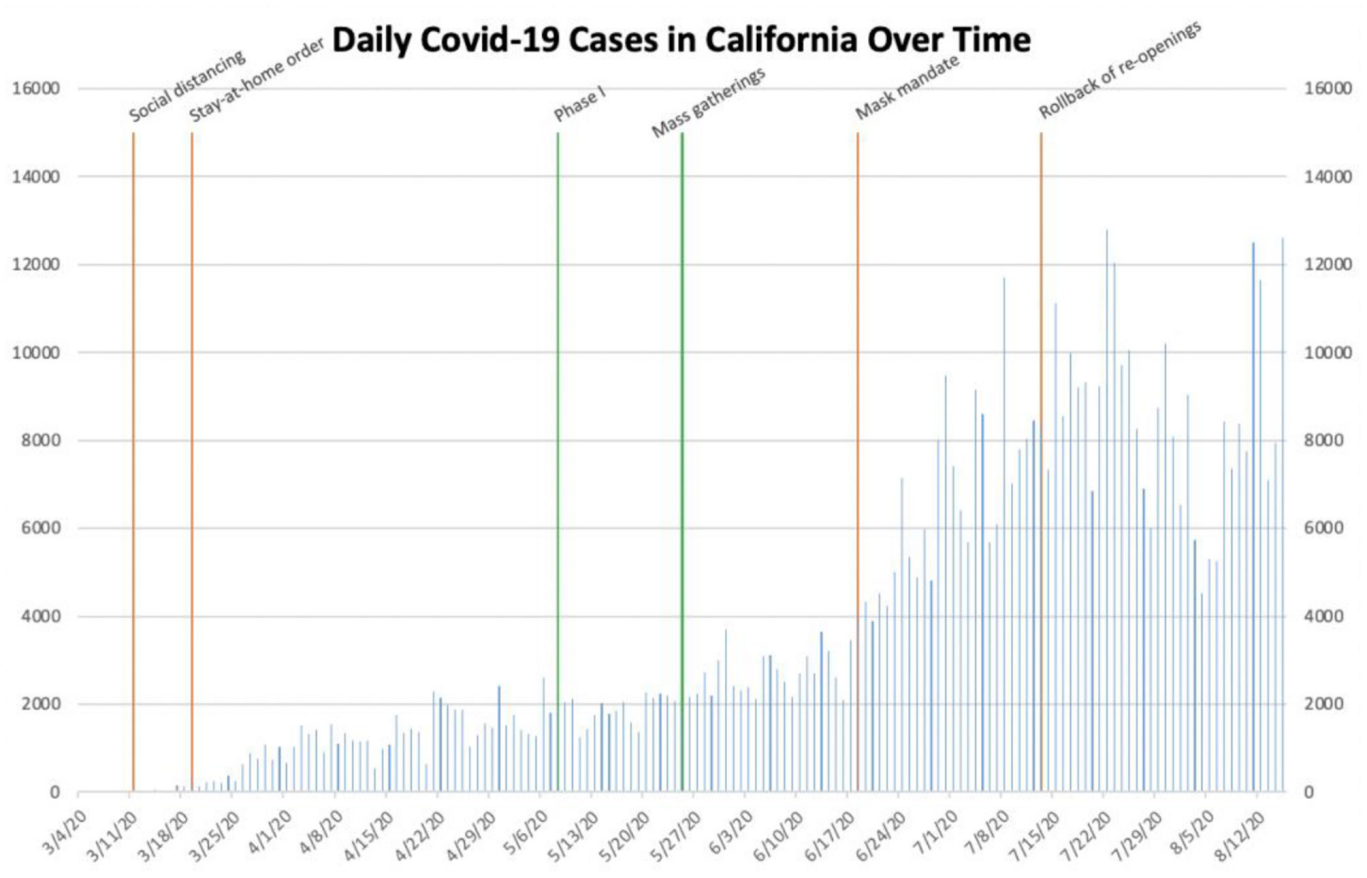
Daily COVID-19 cases in California.

In order to compare New York to other states, we subsequently gathered the same data from Texas, Florida, Louisiana, and California within the same time period (Figures 2–5, respectively) (3). In an attempt to determine the effect of nationwide mass gatherings following the death of George Floyd in Minneapolis, Minnesota, on May 25th on the spread of SARS-CoV-2 within all five states, we also superimposed May 25th onto the domestic epidemiological curves. We similarly hypothesized that the effect of these mass gatherings would begin to be realized after approximately 14 days.

**Figure 6:**
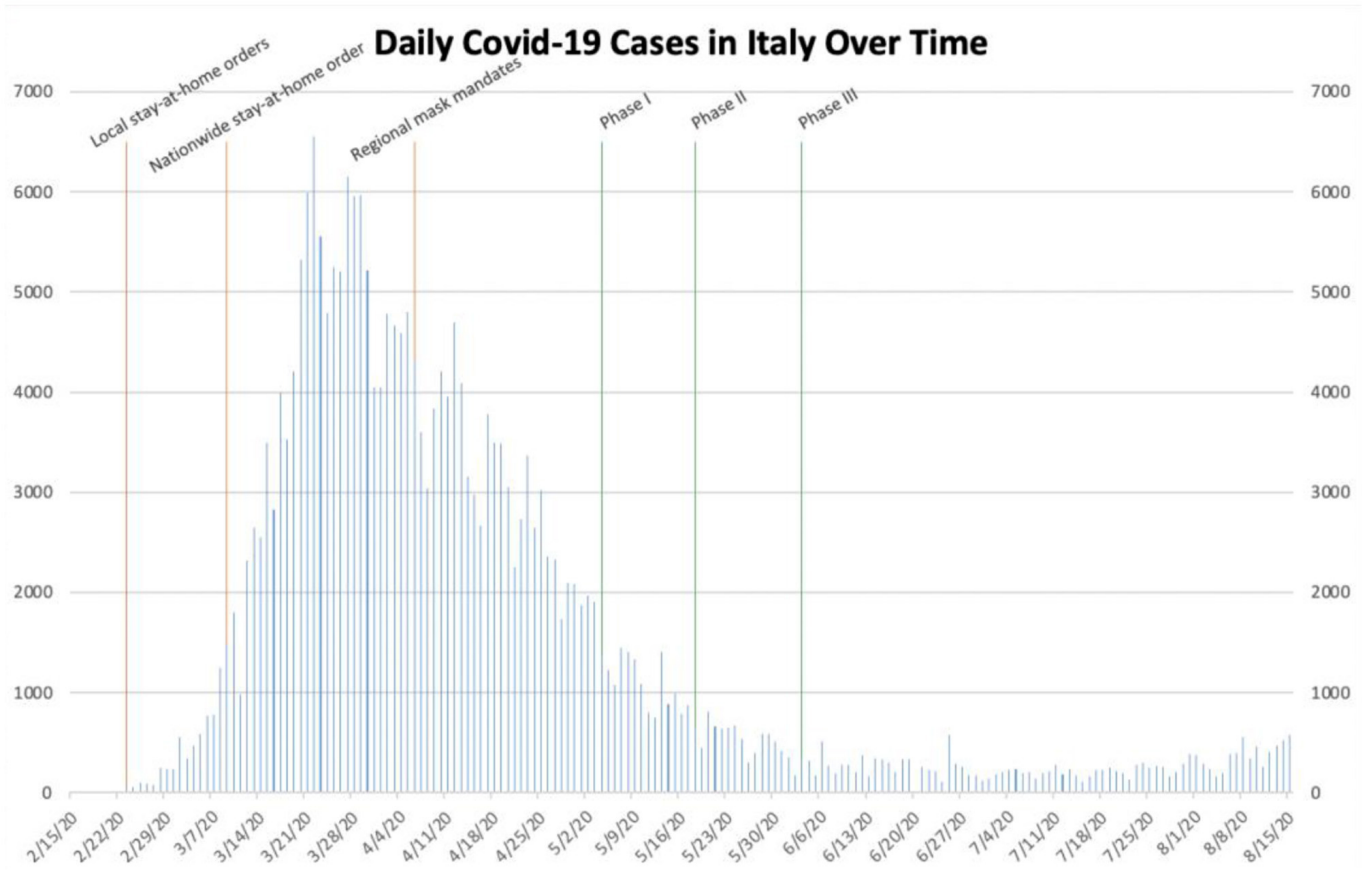
Daily COVID-19 cases in Italy.

**Figure 7:**
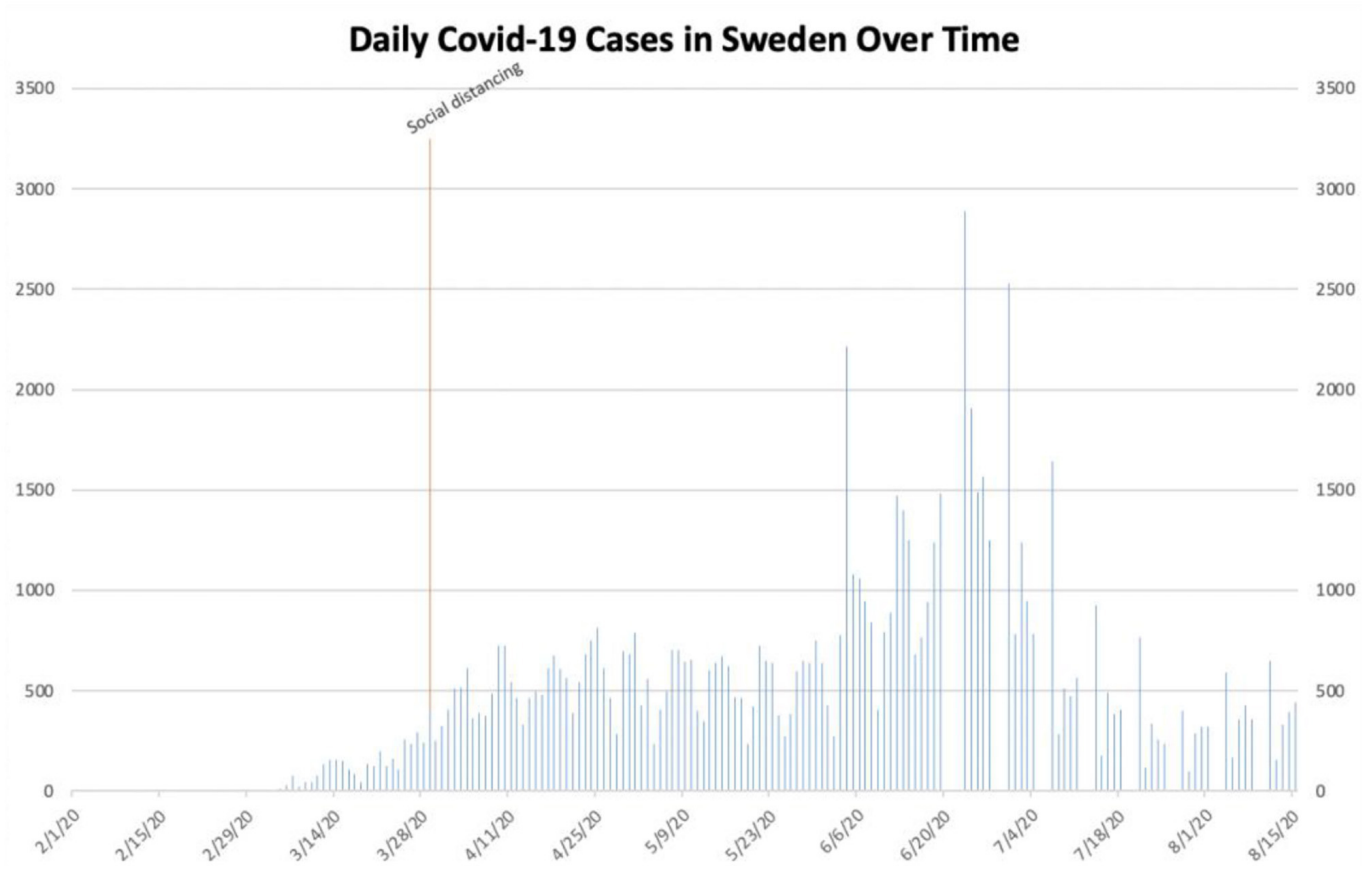
Daily COVID-19 cases in Sweden.

Once these data were collected, we again broadened the scope of the study to compare these states to a few countries around the world. We subsequently collected similar data from Italy (due to its similarity to New York as an early global COVID-19 epicenter; Figure 6) and Sweden (due to its unique response to SARS-CoV-2; Figure 7) (5). The data from these two countries were collected from February 15–August 15, 2020 due to their earlier involvement in the COVID-19 pandemic.

## Results

### New York State

The results of the study varied by location. In New York, Governor Andrew M. Cuomo instituted initial social distancing guidelines on March 12th (6). On March 22nd, Governor Cuomo implemented a stay-at-home order for occupants of the state, excluding only those who had been deemed essential workers (7). While the number of daily cases initially continued to increase in the state following the stay-at-home order, New York soon reached its peak of more than 10,000 daily cases between April 2nd−14th. Following this peak, the number of daily cases declined sharply. As daily cases declined, Governor Cuomo imposed a statewide mask mandate on April 17th (8). Daily cases in the state subsequently decreased to <5,000 cases per day by the end of April. Governor Cuomo then began to implement New York Forward, the stepwise re-opening of non-essential businesses within the state (Table 1) (9). New York Forward officially began on May 15th, when certain regions met specific state guidelines. As the stepwise re-opening of non-essential businesses continued statewide between May 15th and July 20th, the number of daily cases within the state remained <2,000. Even after mass gatherings broke out across the country following the death of George Floyd on May 25th, no resultant surge in daily cases came to fruition within the state.

**Table 1:** Industries affected in various phases of re-opening initiatives.

**Re-opening initiative**	**Location**	**Phase**	**Affected industries (often with capacity restrictions)**
New York forward (9)	New York State	I	Agriculture, construction, manufacturing, retail, wholesale trade
		II	Automobiles, cleaning, food services, offices, personal care, real estate, retail
		III	Food services, personal care
		IV	Arts, education, entertainment, fitness, media production, retail, sports
Open Texas (10)	Texas	I	Arts, entertainment, food services, local government, retail
		II	Arts, entertainment, fitness, food services, local government, offices, personal care, retail, sports
		III	All
Plan for Florida's recovery (11)	Florida	I	Arts, education, entertainment, food services, healthcare, retail, sports
		II	Arts, entertainment, fitness, food services, healthcare, personal care, retail, sports
Open safely (12)	Louisiana	I	Entertainment, fitness, food services, offices, personal care, religion, retail, sports
		II	Entertainment, food services, offices, personal care, sports
Blueprint for a safer economy (13)	California	I	Arts, food services, manufacturing, offices, retail (regional re-openings by county)
N/A (14)	Italy	I	Construction, manufacturing
		II	Arts, entertainment, food services, personal care, religion, retail, travel
		III	Travel

*Industries listed alphabetically within each phase*.

### Texas

In Texas, social distancing was initially implemented on March 19th (15). At this point, the number of daily cases statewide had only reached as high as 34. On April 2nd, as cases began to increase slightly in the state, Governor Greg W. Abbott instituted a statewide stay-at-home order (16). Afterwards, the number of daily cases in Texas remained <2,000 for several months. On April 27th, Phase I of Open Texas, the plan to re-open non-essential businesses within the state, began (Table 1) (10). Phases II and III followed soon after on May 18th and June 3rd, respectively. Between these latter two phases, mass gatherings similarly occurred throughout Texas. After the implementation of Phase III, the number of daily cases within the state began to sharply increase. On June 16th, the number of new daily cases exceeded 4,000; this marked only the second time Texas had exceeded even 2,500 daily cases. In the ensuing weeks, the number of daily cases continued to increase, consistently exceeding 10,000 in the middle of July. As daily cases were still trending upward, Governor Abbott instituted a statewide mask mandate on July 2nd (17). While the number of daily cases continued to increase immediately following this mandate, cases soon plateaued and never once exceeded 10,000 from July 18th through the end of our data collection on August 15th.

### Florida

In Florida, initial social distancing guidelines were implemented on March 17th (18). At that point, the number of daily cases statewide had not yet exceeded 50. On April 1st, as the number of daily cases approached 1,000, Governor Ron D. DeSantis issued a monthlong stay-at-home order (19). Following the implementation of the stay-at-home order, the number of daily cases remained less than 2,000. On May 4th, Florida entered Phase I of its Plan for Florida's Recovery (Table 1) (11). Florida later entered Phase II on June 5th. In between, as in other states, mass gatherings occurred throughout Florida following the death of George Floyd. June 13th, when the number of daily cases first exceeded 2,000, marked the beginning of a statewide outbreak. A steep increase in the number of daily cases led to Governor DeSantis re-closing bars statewide on June 26th in an attempt to slow the then almost-exponential increase of COVID-19 cases (20). Cases subsequently reached their peak on July 12th and then slowly began to decrease again through the end of our data collection.

### Louisiana

Louisiana is somewhat unique among the locations studied due to its two distinct peaks of daily cases between the beginning of March and August 15th. Initial social distancing guidelines in Louisiana were implemented on March 13th, at which point the number of daily cases had yet to surpass 25 (21). As the number of daily cases began to slowly increase, Governor John Bel Edwards issued a statewide stay-at-home order on March 22nd (22). The number of daily cases continued to increase for almost 2 weeks before reaching a peak of more than 2,700 on April 2nd and then decreasing sharply. The number of daily cases then remained <1,500 for almost 3 months. On May 15th, Louisiana entered Phase I of its re-opening initiative (Table 1) (12). As mass gatherings unfolded nationwide beginning on May 25th, the number of daily cases remained <1,500. The state subsequently entered Phase II of its re-opening plan on June 5th. The number of daily cases statewide began to increase soon thereafter, slowly at first and then sharply. As the number of daily cases continued to increase and again exceeded 2,500 on July 11th, Governor Edwards issued a statewide mask mandate (23). The number of daily cases reached its second peak soon thereafter on July 26th and then began to decrease once again.

### California

Initial social distancing guidelines in California were implemented on March 11th (24). On March 19th, Governor Gavin C. Newsom issued the first stay-at-home order in the country (25). The number of daily cases continued to slowly increase for 2 weeks following this proclamation before reaching a steady state of ~2,000 for more than 2 months. On May 8th, as the number of daily cases remained approximately 2,000, California entered Phase I of its Blueprint for a Safer Economy (Table 1) (13). Two and a half weeks later, mass gatherings unfolded throughout the state following the death of George Floyd. Cases then increased slowly for approximately 3 weeks. When the number of daily cases exceeded 4,000 for the first time on June 18th, Governor Newsom immediately instituted a statewide mask mandate (26). Despite the mask mandate, the number of daily cases statewide continued to steadily increase to more than 10,000. On July 13th, Governor Newsom announced the re-implementation of previous restrictions (27). Afterwards, the number of daily cases began to decrease; it reached its nadir of ~4,500 cases on August 4th. However, it then began to rapidly increase once again, exceeding 11,000 several times in the middle of August.

### Italy

Italy was an early global epicenter of the pandemic, recording its first confirmed cases on January 31st (28). On February 23rd, as clusters of cases subsequently emerged in the Lombardy and Veneto regions, stay-at-home orders were instituted in eleven municipalities in northern Italy (29, 30). The number of daily cases nationwide began to increase sharply despite these local, targeted interventions, exceeding 1,000 for the first time on March 8th. On March 9th, Prime Minister Giuseppe Conte expanded the stay-at-home order to the entirety of Italy (31). Immediately following the enactment of the nationwide stay-at-home order, the number of daily cases continued to increase drastically, reaching its peak of more than 6,500 on March 22nd. The number of daily cases then began to decrease precipitously, returning to <1,000 by the middle of May and remaining below that threshold through the end of our data collection on August 15th. On April 6th, as the number of daily cases was already declining considerably, provincial mask mandates were instituted in the regions of Lombardy and Tuscany (32). Cases continued to decrease afterwards. On May 4th, as the number of daily cases nationwide fell below 1,500, Italy entered the first stage of its re-opening initiative (Table 1). It subsequently entered the second stage just 2 weeks later. Then, on June 3rd, Prime Minister Conte announced the easing of domestic travel restrictions, which allowed Italian citizens to travel across regions for the first time since March (14). Despite the easing of these various restrictions nationwide, the number of daily COVID-19 cases remained <700 through August 15th.

### Sweden

Sweden, in contrast to the aforementioned locations, did not enact any true governmental interventions in an effort to stem the spread of SARS-CoV-2. Despite the fact that Sweden reported its first confirmed case of COVID-19 in late January, the Swedish government did not formally recommend social distancing guidelines until March 29th (33, 34). This remained the only domestic measure aimed at stemming the spread of SARS-CoV-2 instituted through at least August 15th. Despite this, the number of daily cases nationwide remained <1,000 until June 4th. The country reached its peak of almost 2,900 daily cases soon after, on June 23rd. The number of daily cases returned to <1,000 after approximately 4 weeks and remained that way through August 15th.

## Discussion

Based on the data collected, it is reasonable to conclude that governmental interventions, especially stay-at-home orders and mask mandates, have been successful to varying degrees in stemming the spread of SARS-CoV-2 within these different locations. In New York, the stay-at-home order seemingly helped to drastically decrease the number of daily cases during the early outbreak, while a mask mandate and social distancing measures likely helped prevent a future outbreak, even after businesses re-opened and mass gatherings took place (Figure 1). In Texas, Phase III of Open Texas and the July 4th holiday weekend likely both contributed to the subsequent surge in the number of daily cases. The mask mandate seemingly helped slow the spread of SARS-CoV-2 later, in the midst of an outbreak (Figure 2). In Florida, Phase II of its re-opening initiative likely contributed to the ensuing outbreak, as the number of daily cases increased from <2,000 to more than 10,000 within the subsequent month. The June 26th re-imposition of restrictions on bars statewide seemingly helped control the outbreak, as cases began to sharply decrease approximately 2 weeks afterwards (Figure 3). Since Florida never instituted a statewide mask mandate, it is impossible for us to determine the effect of sporadic, optional mask wearing throughout the state. The stay-at-home order in Louisiana likely helped reverse a sharp early increase in daily cases, as a resultant decline was seen after approximately 10 days. After Phase II of its re-opening initiative was implemented in early June, cases increased from 427 to more than 1,300 within 3 weeks and continued to increase to more than 3,800 by the end of July. The mask mandate implemented on July 11th ostensibly helped reverse this trend, as cases reached a peak 15 days later and then began to decrease sharply (Figure 4). When Italy was an early global COVID-19 epicenter, a nationwide stay-at-home order likely contributed to the drastic reduction in the number of daily cases that began 13 days later. It is also possible that previously implemented provincial mask mandates and social distancing measures helped prevent a second widespread outbreak within the country through the middle of August (Figure 6).

California and Sweden, in contrast to the aforementioned locations, present somewhat confounding data on the effect of governmental interventions on the spread of SARS-CoV-2. California, which instituted governmental interventions similar to other states', did not demonstrably gain substantial benefit from either the imposition of a statewide mask mandate or the re-institution of restrictions on certain industries. Daily cases continued to increase in the state despite the mask mandate, while the re-implementation of previous restrictions seemingly only contributed to a transient decrease in the number of daily cases. Phase I of its re-opening initiative and mass gatherings also seemingly did not contribute meaningfully to the state's outbreak, as cases began to increase sharply at least 3 weeks after each of them began (Figure 5). Sweden remains an enigma in its fight against SARS-CoV-2. Due to its unique hands-off approach, it would have been expected to experience rampant, uncontrolled spread of SARS-CoV-2. However, its lone outbreak of COVID-19 during the time period studied abated relatively quickly despite no significant governmental intervention (Figure 7). One possible explanation for this is the fact that Sweden has a lower population density than the other locations studied (Sweden's population density: approximately 64 inhabitants per square mile; Louisiana: 105; Texas: 110; California: 254; Florida: 397; New York State: 421; Italy: 533) (5). While population density undoubtedly impacts the transmissibility of SARS-CoV-2, it is unclear to what extent it does so. Additionally, unlike the United States', Sweden's population is one of greater cultural homogeneity; this makes comparisons between the two nations extremely difficult. Furthermore, Sweden fared significantly worse with respect to COVID-19 hospitalizations and deaths than other Scandinavian countries which are more culturally similar to it, possibly due to the fact that these nations implemented more stringent interventions (35). It is thus important to note that Sweden's model should likely not be replicated in other countries around the world.

This study has inherent limitations, such as not accounting for either the extent to which various populations adhered to respective governmental interventions or the differences in viral transmission among various settings within a single state or country (e.g., urban, suburban, rural). In addition, there are differences in how cases of COVID-19 are defined and counted in various locations, as well as inconsistencies in the reporting of these data. Still, we believe that this study demonstrates the efficacy of governmental interventions in stemming the spread of SARS-CoV-2. Though admittedly burdensome, temporary stay-at-home orders proved quite efficacious in drastically decreasing the number of daily cases within a given location, especially during outbreaks. Social distancing measures and mask mandates, meanwhile, often proved valuable in helping to prevent subsequent outbreaks.

Through identification of specific governmental interventions that have been most effective, this research has the potential to help guide future governmental responses to the COVID-19 pandemic. Due to their high economic and psychological costs to a population, temporary stay-at-home orders should likely be used only to control an ongoing outbreak of COVID-19 within a given location. Social distancing measures and mask mandates, meanwhile, have proven to be effective in preventing subsequent outbreaks. Additionally, these measures come at much lower economic and psychological costs to a population. Thus, the precautionary principle strongly suggests that these interventions, at a minimum, are worthwhile and advisable.

## Author Contributions

JM, AM, and LT were responsible for data collection. JM was responsible for writing of manuscript. MW was responsible for developing idea for manuscript and for editing and overall guidance regarding direction of paper. All authors contributed to the article and approved the submitted version.

## Conflict of Interest

The authors declare that the research was conducted in the absence of any commercial or financial relationships that could be construed as a potential conflict of interest.

## Publisher's Note

All claims expressed in this article are solely those of the authors and do not necessarily represent those of their affiliated organizations, or those of the publisher, the editors and the reviewers. Any product that may be evaluated in this article, or claim that may be made by its manufacturer, is not guaranteed or endorsed by the publisher.
